# Implementation of integrated supportive supervision in the context of coronavirus 19 pandemic: its effects on routine immunization and vaccine preventable surveillance diseases indicators in the East and Southern African countries

**DOI:** 10.11604/pamj.2021.38.164.27349

**Published:** 2021-02-12

**Authors:** Isah Mohammed Bello, Emmaculate Lebo, Messeret Eshetu Shibeshi, Godwin Ubong Akpan, Jethro Chakauya, Balcha Girma Masresha, Fussum Daniel

**Affiliations:** 1 World Health Organization, Inter-Country Support Team Office for East and Southern Africa, P.O. Box 5160, Harare, Zimbabwe,; 2 World Health Organization, Regional Office for Africa, Cité du Djoué, Brazzaville, Congo

**Keywords:** Integrated supportive supervision, COVID-19, routine immunization, vaccine preventable diseases, surveillance, East and Southern Africa

## Abstract

**Introduction:**

the coronavirus disease (COVID-19) global pandemic has caused serious disruption to almost all aspect of human endeavor forcing countries to implement unprecedented public health measures aimed at mitigating its effects, such as total lockdown (inter and intra), travel bans, quarantine, social distancing in an effort to contain the spread of the virus. Supportive supervision is a functional component of the immunization systems that allows identification of existing gaps, provides an opportunity for onsite training, and document real-time findings for improvement of the program. The control measures of COVID-19 pandemic have also resulted in limitation of operations of the immunization system including supportive supervision. This has limited many aspects of supportive supervision for surveillance and routine immunization monitoring system in the East and Southern African countries. The aim of this study is to identify the effects of COVID-19 on Integrated Supportive Supervision visits for expanded programme on immunization (EPI) and how it influences the immunization and vaccine preventable disease (VPD) surveillance indicators, and its short-term effect towards notification of increase or decrease morbidity and mortality.

**Methods:**

we reviewed the integrated supportive supervision (ISS) data and the routine administrative coverage from 19 countries in the East and Southern Africa (ESA) for the period January to August 2019 to analyze the trends in the number of visits, vaccine-preventable diseases (VPD), and routine immunization (RI) indicators using t-test, and compare with the period January to August 2020 during the months of the COVID-19 pandemic.

**Results:**

thirteen countries out of the 19 considered, had shown a decline in the number of integrated supportive supervision (ISS) visits, with 10 (77%) having more than 59% decrease during the January-August 2020 as compared to the same period 2019. Eleven (57%) of the countries have shown a decrease (p-value < 0.05). Ethiopia and Kenya had the highest drop (p-value < 0.000). Six (32%) had an increase in the number of visits, with Madagascar, Zambia, and Zimbabwe having >100% increase in the number of visits. Sixty-seven percent (67%) of the countries that have decreased in the number of ISS visits have equally witnessed a drop in DPT3 administrative coverage. Countries with a low proportion of outreach sessions conducted in the period of January - August 2020, have all had sessions interruption, with more than 40% of the reasons associated with the lockdown.

**Conclusion:**

countries have experienced a decrease in the number of supportive supervision visits conducted, during the period of the COVID-19 pandemic and, this has influenced the routine immunization and vaccine-preventable diseases surveillance (VPD) process indicators monitored through the conduct of the visits. Continuous decrease in these performance indicators pose a great threat to the performance sustained and the functionality of the surveillance and immunization system, and consequently on increased surveillance sensitivity to promptly detect outbreaks and aiming to reducing morbidity and mortality in the sub-region.

## Introduction

In 2015, member states in the African region under the leadership of the World Health Organisation (WHO) and other stakeholders adopted a strategic plan on immunization (2014-2020), which is aimed at improving immunization coverage. It was built on the lessons learned from the 2009-2013 Global Immunization Vision and Strategy (GIVS) framework and aligned with the Global Vaccine Action Plan (GVAP), guiding regional and global response to address immunization gaps, interrupt the transmission of poliovirus, and achieve measles elimination and the control of other vaccine preventable diseases [1].

According to WHO, the number of infants who did not receive the third dose of DTP vaccines in 2014 in the region was estimated to be around 7.4 million out of an annual birth cohort of 32.7 million representing 23%. The coverage has remained stagnant at around 70% for a long period of time [1]. Some of the challenges that the immunization programme is facing includes lack of adequate and sustainable resources, logistics and laboratory facilities, data quality and frequent vaccine stock out [2]. Several countries in the region had come up with annual and operational plans, which are geared towards improving coverage at both national and subnational levels in order to interrupt poliovirus, certification and close immunity gaps including mitigation measures taken guided by periodic risk assessments. Consequently, outbreaks of vaccine preventable diseases (VPD) like Yellow Fever, Lassa Fever, Ebola etc. have had an adverse effect on the performance of the health system in general, thereby affecting both access and utilization as well as disruption of the health service delivery in some African countries [3]. Measles outbreaks are mostly occurring in communities with low or partially immunized children [4], which are communities with low or no herd immunity strong enough to prevent disease transmission in these areas [5]. One of the approaches adopted in African region is the adoption and use of the Reaching Every District (RED), which has played an important role in strengthening the immunization systems to achieve equity and increasing coverage. The RED strategy is built upon five (5) key principles that include planning and management of resources, community engagement, supportive supervision, reaching all eligible and target population, monitoring and use of data for action [6].

Supportive supervision (SS) as one of the strategies of RED has continued to attract attention as an effective tool in managing and improving the quality of health services [6], it is seen to be an effective strategy towards promoting the delivery of public health services and the quality of health system at all levels through development and increasing the knowledge and competence of health care workers [7]. SS help tightens the relationships that exist in a system, with emphasis on identifying and resolving problems onsite, as well as optimizing the allocation of resources and promoting teamwork and communication [8]. The World Health Organization had on March 2020 declared an outbreak of the coronavirus (COVID-19) disease as a global pandemic, which is a public health problem that is threatening the entire world [9, 10]. COVID-19 is a contagious disease that is rapidly spreading from Wuhan City, Hubei Province of China to the rest of the world. Globally, as of November 11^th^ 2020 a total of 50,810,763 confirmed cases of COVID-19, including 1,263,844 deaths have been reported [11]. In Africa, all the countries in the region had been affected with a cumulative total number of 1,363,929 confirmed cases and 30,883 deaths [12].

Countries had adopted several strategies in trying to reduce the spread of the disease and breaking the chain of transmission as a standard precautionary measure for fighting the COVID-19 pandemic, through the application of strict control measures, which includes total lockdown, (intra and inter), social distancing among others, and that had led to disruption of routine preventive and curative health services among others [13]. The routine immunization programme had been affected due alleged fears of contracting the disease in health facilities, staff shortage due to reassignment of health care workers to support the COVID-19 outbreak response, stock-out of vaccines and other supplies, leading to partial or complete closure of some health facilities. This also includes planned mass vaccination campaign for vaccine preventable diseases (VPD) in some countries that had to be postponed [14]. Fear of contracting the virus, with a significant shift in parents and community behaviour due to social distancing are forcing parents and caregivers to hesitate to go for routine immunizations [15].

It becomes imperative to understand the effects of COVID-19 on basic routine immunization services as it continued to be disrupted, especially with the impending warning from health experts on the possible resurgence of Polio and VPD outbreaks in polio-free countries [16]. The World Health Organisation (WHO) had also developed guidance for countries as measures for preventing COVID-19 transmission in health care settings, in an effort to ensure the continuity of basic essential health services, including immunization [17, 18]. In the African region, supportive supervision is conducted using mobile phone-based data collection with the Open Data Kit (ODK) application, which started in Nigeria in 2015 and was then rolled out to other countries of the region [19]. This paper examines the effects of the COVID-19 pandemic on the performance of the ISS in the 19 countries of East and Southern African Subregion (ESA) by comparing the number of ISS visits and some VPD and routine immunization (RI) indicators in the first 3 quarters of 2020 (COVID-19 pandemic) and same period in 2019 (before the COVID-19 pandemic).

## Methods

Data stored on the server for 19 countries in the ESA sub-region implementing the integrated supportive supervision was used. The subregion is a mixture of countries with different population size, having both fragile and weak health system and those with matured health system and sustained high coverage [20]. Monthly routine administrative immunization data was also used for the same period January to August 2019 and 2020. The data was prepared for analysis in Microsoft Excel, with the domain of analysis being country level. Prepared data was analysed in SPSS to assess the level of significance differences, while some of the data sets were analysed in excel. We reviewed the number of integrated supportive supervision (ISS) visits for the Expanded Programme on Immunization (EPI) programme for the period January to August 2019/2020 to compare the trends in the number of ISS visits conducted monthly. We also analysed some selected indicators that are being monitored on monthly basis for vaccine preventable diseases (VPD) and routine immunization (RI) before and after the onset of the COVID-19 pandemic using paired t-test comparing both absolute values and proportions.

The indicators considered for VPD surveillance are Proportion of Health Facilities visited with Good knowledge of Measles Case Definition and the Number of suspected unreported AFP and measles cases found during the visit, while the indicators considered for routine immunization (RI) are, the proportion of health facilities (HF) visited with reaching every district (RED) microplan available in 2019.

The ISS data is collected using a standard checklist, which was designed to collect information on vaccine preventable disease (VPD) surveillance and routine immunization process indicators at the health facility with additional features of coordinates location, in-built features for easy flow of questions, pictures for validation etc. A Power-BI dashboard was linked to the ISS checklist on the ODK to fetch the data in real time, which allow program managers monitor the performance of the programme. ISS visits are conducted at different frequency depending on the country context (Weekly, Bi-Weekly, Monthly and Quarterly).

## Results

The status of COVID-19 reported cases and deaths based on reports received from countries through the World Health Organization (WHO) emergency programme as depicted in Table 1, showed the level of transmission as at the end of August 2020, the tables depicts that three (3) countries have sporadic cases, four (4) countries have cluster of cases while the remaining twelve (12) countries are having active community transmission. South Africa has the highest cumulative incidence of COVID-19 per 1 million population of 10,497, while Eritrea and Seychelles have 0. The number of integrated supportive supervision (ISS) visits has dropped from 34,983 in January - August 2019 to 26,691 in the same period of 2020, representing a 24% drop. Thirteen (13) countries (68%) have witnessed a decrease in the number of visits as shown in Table 2, while 6 (32%) have an increase in the number of visits. Madagascar, Zambia and Zimbabwe have > 100% increase in the number of visits. Seven countries showed difference in the number of visits made (p < 0.05), with the most difference noted in Ethiopia (p-value = 0.001), Kenya (p-value = 0.000, Mauritius (p-value = 0.003) and Uganda (p-value = 0.002).

**Table 1:** COVID-19 incidence and transmission status in East and Southern African (ESA) countries as of end August 2020

Country	Estimated Total Population	Cumulative COVID cases	Cumulative Cases per 1 million Population	Cumulative Deaths	Cumulative Deaths per 1 million Population	Transmission Classification
Botswana	2,351,627	1,633	694	6	3	Cluster of Cases
Eritrea	3,546,421	318	90	0	<1	Sporadic Cases
Eswatini	1,160,164	4,510	3887	91	78	Community transmission
Ethiopia	114,963,588	49,654	432	770	7	Community transmission
Kenya	53,771,296	33,794	628	572	11	Community transmission
Lesotho	2,142,249	1,066	498	31	14	Cluster of Cases
Madagascar	27,691,018	14,791	534	190	7	Community transmission
Malawi	19,129,952	5,528	289	174	9	Community transmission
Mauritius	1,271,768	346	272	10	8	Sporadic Cases
Mozambique	31,255,435	3,760	120	22	1	Community transmission
Namibia	2,540,905	7,116	2,801	69	27	Community transmission
Rwanda	12,952,218	3,843	297	16	1	Cluster of Cases
Seychelles	98,347	131	1,332	0	<1	Sporadic Cases
South Africa	59,308,690	622,551	10497	13981	236	Community transmission
South Sudan	11,193,725	2,519	225	47	4	Community transmission
Tanzania	59,734,218	509	9	21	<1	Community transmission
Uganda	45,741,007	2,756	60	28	1	Cluster of Cases
Zambia	18,383,955	11,902	647	284	15	Community transmission
Zimbabwe	14,862,924	6,406	431	196	14	Community transmission

**Table 2:** comparison between number of Integrated Supportive Supervision (ISS) visits (January - August 2019/2020)

Countries	Supportive Supervision (ISS) Visits		Test Statistic	95% Confidence Interval of the Difference		P-value
	Jan-Aug 2019	Jan-Aug 2020		Upper	Lower	
Botswana	73	91	-0.84	-13.843	11.843	0.859
Eritrea	190	46	1.635	-6.691	36.691	0.146
Eswatini	162	55	2.725	1.621	22.879	0.030
Ethiopia	5,030	3,260	5.1	118.944	324.556	0.001
Kenya	9,909	2,558	7.189	550.698	1090.552	0.000
Lesotho	5	0	1.256	-.552	1.802	0.250
Madagascar	2,427	5,761	-2.304	-154.008	2.008	0.055
Malawi	834	247	2.396	.818	125.932	0.048
Mauritius	52	5	4.374	2.354	7.896	0.003
Mozambique	917	174	2.074	-8.520	130.270	0.077
Namibia	194	121	1.027	-16.432	41.682	0.338
Rwanda	61	67	-0.96	-12.811	11.811	0.926
Seychelles	14	12	1.515	-.631	2.881	0.174
South Africa	465	206	1.867	-7.589	64.589	0.104
South Sudan	6,066	8,640	3.312	22.061	132.189	0.013
Tanzania	1,758	795	1.88	-31.599	276.599	0.102
Uganda	6,173	2,830	4.689	150.884	457.866	0.002
Zambia	632	1,702	-2.056	-166.382	11.632	0.079
Zimbabwe	21	121	-2.621	-21.162	-1.088	0.034
Total	34,983	26,691	4.629	47.661	118.642	0.000

The VPD surveillance indicator, which looks at the proportion of health care workers with adequate knowledge of Acute Flaccid Paralysis (AFP) and measles case definition in the health facilities visited in the period January - August 2019 showed a decline in 9 (47%) and 13 (68%) out of the 19 countries respectively (p-value < 0.05) as compared to January - August 2020. Nine (9) and Four (4) countries representing 47% and 32% respectively have either increase or have equal the proportion when compared to same period 2019 and 2020, while Lesotho did not conduct any ISS visit in the period January - August, 2020 (Table 3). The number of unreported suspected AFP and measles cases found during the ISS visits also dropped from 222 and 1,419 records in the period January - August 2019 to 157 and 500 in 2020 same period respectively. More so, measles suspected unreported cases have shown >50% decrease in five (5) countries, with four (4) countries having percentage increase of more than 30% when compared to same period, four (4) countries did not report any case, while 1 country (Zimbabwe) have cases reported in January - August 2020 only, with no case reported in the period January - August 2019. Conversely the number of suspected unreported AFP cases has equally dropped from 222 to 157 when comparing data for the period January - August 2019/2020. Only four (4) countries have reported more cases in 2020 as compared to 2019 for the period January - August (Table 3).

**Table 3:** vaccine preventable disease (VPD) indicators comparison (January - August 2019/2020)

Countries	Number of ISS Visits		Proportion of HF FP with Knowledge of AFP Case Definition			Proportion of HF FP with Knowledge of Measles Case Definition			Unreported Suspected Measles Cases - 2019		Unreported Suspected AFP Cases - 2020	
	Jan - Aug 2019	Jan - Aug 2020	Jan - Aug 2019	Jan - Aug 2020	p-value	Jan - Aug 2019	Jan - Aug 2020	p-value	Jan - Aug 2019	Jan - Aug 2020	Jan - Aug 2021	Jan - Aug 2022
Botswana	73	91	86%	91%	0.3149	92%	77%	0.0100	1	1	1	1
Eritrea	190	46	98%	98%	1.0000	95%	100%	0.1224				
Eswatini	162	55	79%	84%	0.4219	88%	75%	0.0209	6	2		
Ethiopia	5,030	3,260	93%	88%	0.0001	96%	92%	0.0001	70	59	10	30
Kenya	9,909	2,558	90%	90%	0.0001	93%	92%	0.0812	96	12	30	12
Lesotho	5	0	80%			100%				0		
Madagascar	2,427	5,761	94%	95%	0.0650	94%	96%	0.0001	281	47	8	30
Malawi	834	247	87%	77%	0.0001	95%	88%	0.0001	19	116	18	9
Mauritius	52	5	96%	100%	0.6516	98%	80%	0.0402				
Mozambique	917	174	89%	89%	1.0000	79%	71%	0.0201	70	46	11	1
Namibia	194	121	81%	85%	0.3638	93%	86%	0.0414	7	7	1	4
Rwanda	61	67	93%	94%	0.8191	93%	82%	0.0635	11	22		
Seychelles	14	12	86%	75%	0.4853	86%	100%	0.1862				
South Africa	465	206	88%	88%	1.0000	94%	94%	1.0000	3	4	42	15
South Sudan	6,066	8,640	85%	86%	0.0892	93%	71%	0.0001	82	56	40	28
Tanzania	1,758	795	82%	86%	0.0122	75%	75%	1.0000	237	62	12	6
Uganda	6,173	2,830	72%	76%	0.0001	87%	86%	0.1948	535	52	42	13
Zambia	632	1,702	98%	79%	0.0001	98%	89%	0.0001	1	9	7	8
Zimbabwe	21	121	76%	91%	0.0451	90%	68%	0.0405		5		
Grand Total	34,983	26,691	86%	87%	0.0003	92%	77%	0.0001	1,419	500	222	157

Health workers´ knowledge on AFP and Measles case definition was discordant in seven countries, with the remaining being equal. Not much difference was noted on the health workers knowledge on AFP in Madagascar when two years were compared (p-value = 0.065), whereas for measles, a noticeable difference was observed (p-value = 0.001). Ethiopia, Uganda and Zambia have knowledge on both AFP and measles being the same (p-value = 0.0001) (Table 3). The proportion of health facilities with RED microplan available showed a drop (p-value < 0.05) in 12 (63%) countries in January - August 2020 when comparing with 2019. Six (6) countries representing 32% have an increase in 2020 when comparing the data for same period January - August 2019 (p-value >0.05). One country (Lesotho) did not have any single visit in the period under review (Table 4).

**Table 4:** routine immunization (RI) indicators comparison (January - August 2019/2020)

Countries	Total ISS visit to Unique HFs		% HF with RED Plan Available			% Fixed Sessions Conducted			% Outreach Sessions Conducted			% HF with Updated Monitoring Chart		
	Jan - Aug 2019	Jan - Aug 2020	Jan - Aug 2019	Jan - Aug 2020	P-Value	Jan - Aug 2019	Jan - Aug 2020	P-Value	Jan - Aug 2019	Jan - Aug 2020	P-Value	Jan - Aug 2019	Jan - Aug 2020	P-Value
Botswana	66	74	18%	12%	0.3204	90%	90%	1.0000	87%	41%	0.0001	14%	5%	0.0674
Eritrea	147	27	44%	48%	0.7016	98%	100%	0.2232	67%	100%	0.0001	67%	41%	0.0022
Eswatini	144	46	9%	2%	0.1132	98%	99%	0.6541	50%	31%	0.0246	30%	13%	0.0223
Ethiopia	3,054	1,280	62%	33%	0.0010	98%	96%	0.0020	96%	95%	0.1386	45%	22%	0.0001
Kenya	7,883	1,318	18%	16%	0.0784	91%	92%	0.2369	62%	63%	0.4884	32%	22%	0.0001
Lesotho	5	0	40%			81%			94%			80%		
Madagascar	1,362	1,970	28%	31%	0.0627	85%	89%	0.0006	81%	71%	0.0001	39%	29%	0.0001
Malawi	710	203	61%	60%	0.7970	99%	98%	0.2531	97%	96%	0.4770	38%	33%	0.1932
Mauritius	45	4	27%	25%	0.9318	75%	100%	0.2595	93%	0%	0.0001	42%	25%	0.5114
Mozambique	638	151	42	30%	0.0068	93%	99%	0.0048	62%	79%	0.0001	39%	37%	0.6501
Namibia	171	70	12%	24%	0.0197	98%	99%	0.5874	74%	88%	0.0173	31%	26%	0.4409
Rwanda	61	65	8%	20%	0.0547	98%	100%	0.2538	94%	100%	0.0459	41%	32%	0.2958
Seychelles	14	5	43%	0%	0.0842	100%	100%		100%	100%		21%	20%	0.0963
South Africa	385	157	2%	5%	0.0579	63%	75%	0.0073	91%	100%	0.0001	17%	25%	0.0325
South Sudan	2,461	1,844	41%	23%	0.0001	98%	95%	0.0001	75%	70%	0.0003	27%	25%	0.1395
Tanzania	1,509	529	25%	26%	0.6488	92%	97%	0.0001	78%	77%	0.6343	15%	14%	0.5768
Uganda	3,573	1,138	38%	29%	0.0001	95%	89%	0.0001	89%	83%	0.0001	21%	14%	0.0001
Zambia	408	1,027	34%	12%	0.0001	100%	94%	0.0001	99%	76%	0.0001	38%	10%	0.0010
Zimbabwe	21	110	19%	55%	0.0026	92%	96%	0.4264	28%	46%	0.1284	62%	69%	0.5306
Grand Total	22,657	10,018	33%	26%	0.0001	96%	94%	0.0001	83%	78%	0.0001	31%	22%	0.0001

Generally, the proportion of HF with RED micro-plan was low across all the countries with an average of 30% in 2019 and 25% in 2020 January - August. The proportion of session conducted against planned for both fixed and outreach session also depicts that 9 (47%) of the countries have decreased for fixed session during the period January - August 2019 against 2020, nine (9) countries have increased, 1 country (Lesotho) did not record any ISS visit in the period January - August 2020 (Table 4). There was also a corresponding decrease in 12(63%) of the countries for outreach session planned when comparing January-August 2019 and 2020, with 7(32%) of the countries having an increase in the proportion (p-value > 0.05), one country (Lesotho) did not report any visit in the period January - August 2020. One of the countries (Seychelles) have both achieved 100% in both periods under consideration for both fixed and outreach sessions planned (Table 4).

**Figure 1:**
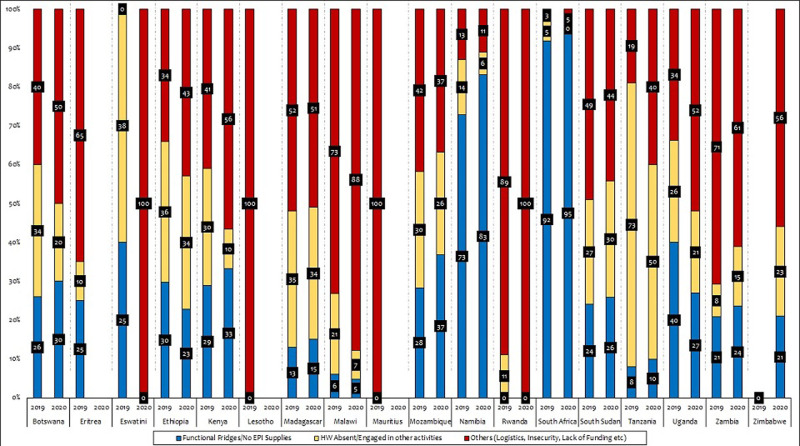
reasons for session interruption (January - August 2019/2020)

Regarding the proportion of health facilities (HFs) with updated monitoring chart, 11 (58%) of the countries witnessed a statistically significant reduction (p-value < 0.05) for the entire period January - August 2010 as compared to January - August 2019 (Table 4). All countries have reported session interruption with reasons in the period January - August 2019 except for Zimbabwe, who reported session interruption, without indicating the reasons for interruption. Eight (8) of these countries representing 43% have >50% of the reasons for interruptions as others (logistics, insecurity, lack of funding etc.). In 2020 same period, fifteen (15) countries have indicated session interruption with reasons, while 5 countries did not indicate the reasons for interruption. Eight (8) countries representing 53% have reasons for session interruption as others (logistics, insecurity, lack of funding and lockdown etc.), the other reasons for session interruption are related to availability of functional fridges / EPI supplies and health workers having 33% and 37% for 2019, and 27% and 31% for 2020 respectively (Figure 1).

**Figure 2:**
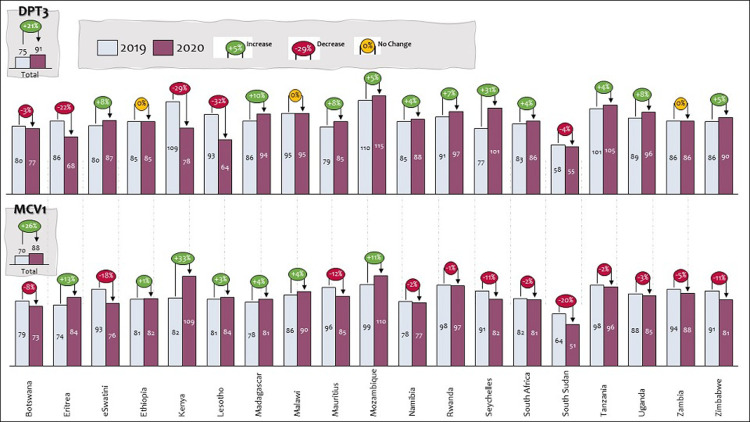
routine immunization (RI) administrative coverage (January - August 2019/2020)

The routine immunization coverage also depicts that 7 (37%) countries have increased administrative coverage for DPT3 containing vaccine, 10 (53%) countries have witnessed drop in coverage, and 2 (11) countries have maintained the same coverage when comparing the period January - August 2019/2020. In contrast, 6 (32%) of the countries have an increasing coverage for Measles Containing Vaccine (MCV1), while the remaining 13 (68%) have decrease in coverage using administrative data comparing the period January - August 2019 and 2020 respectively (Figure 2).

## Discussion

In this study, we have found out that the situation in the countries across the sub-region varies considerably, with decrease in the number ISS visits during the early period of COVID-19 pandemic in 13 countries. Five (5) countries (Ethiopia, Kenya, Madagascar, South Sudan and Uganda) contributed more than 85% of the visit in January - August 2019, but only two (2) countries (Madagascar and South Sudan) have shown an increase in the period January - August 2020, with Ethiopia, Kenya and Uganda having shown a decline of greater than 35%, this also correlate with the incidence of COVID-19 cases in Ethiopia and Kenya who are having community transmission and having the highest number of reported cases in the sub-region aside from South Africa. However, the increase in the number of ISS does not necessarily translate to improvement in all VPD surveillance indicators. All the six (6) countries that have increased number of visits, have witnessed a corresponding increase in knowledge of AFP case definition except Zambia, whereas all the countries showed decline in knowledge of measles case definition. On the other hand, only three (3) countries (Kenya, Mozambique and Tanzania) with an increase in the proportion of fixed and outreach session conducted have a corresponding increase in DPT3 and MCV1 coverage, this shows that there is direct relationship between the increase in the proportion of session conducted and the outcome (immunization coverage), similar to findings that link conduct of sessions to increasing coverage [21-23]. Four (4) countries (Madagascar, South Sudan, Zambia and Zimbabwe), with ≥ 30% increase in number of visits when compared with last year same period (January - August, 2019/2020) representing 67% have community transmission, while the remaining two countries (Botswana and Rwanda) having only 20% and 9% increase respectively have cluster of cases. This indicates a programmatic decision to maintain ISS visits and support to maintain essential health services even when intensive COVID transmission continued [24, 25].

Similarly, the study also found that eight (8) countries representing 67% that have decrease in the number of ISS visits have equally witnessed a drop in DPT3 coverage when comparing the period January - August 2019 and 2020, signifying a relationship in the effects of the visits on the outcome indicator (DPT3) as evidence from a similar study in India, which suggest that supportive supervision improves immunization coverage, and equally serves as a good tool for strengthening health system at the local level [26]. It also support the fact that increased number of ISS visits does not necessarily improve immunization coverage as all the countries with increased visits in period January - August 2019 as against January - August 2020, the administrative coverage has dropped except in Botswana and South Sudan for DPT3 only, this is in agreement with studies that defies linking supportive supervision with direct positive effects to outcome [27, 28]. It was also noted that countries with low proportion of outreach sessions conducted in the period January - August 2020, have all had session interruption, with more than 40% of the reasons associated with lockdown. However, other reasons for session interruption (lack of functional fridges/EPI supplies and health workers availability) also constitutes more 50% of the reasons, with two countries (Namibia and South Africa) having more than 80% in both periods under consideration (2019/2020), indicating systemic issues with the immunization system in these countries. Studies suggests that reasons for session interruption differ from country to country and these factors include among others: parent/caregivers’ attitude, logistics, health workers knowledge and availability etc. that are affecting immunization service delivery [29, 30].

The reduction in the number of ISS visits, which gave rise to a reduction in the number of unreported suspected AFP and measles cases during the period January - August 2020 as compared to same period of 2019 increases the likelihood of missing possible outbreak of both diseases, the possibility of having outbreaks cannot be overruled. In addition to the drop in MCV1 across 12 (63%) countries in the sub-region during the period of January - August 2019 coupled with reduction in the number of fixed and outreach session, in addition to the disruptions in routine immunization services, the chances of having accumulation of susceptible young children, who will be at risk of causing measles outbreaks is high [31, 32]. In the midst of the COVID-19 pandemic, basic health services had been disrupted and the World Health Organization (WHO) have released guidance to support countries on ensuring the continuity of basic health services, while at the same time maintaining all the necessary COVID-19 infection and prevention control (IPC) measures [18]. Countries could explore other opportunities of maintaining supportive supervision in the context of the pandemic through the use of virtual remote supportive supervision, when physical (in-person) visits are not feasible due to the coronavirus pandemic. This will ensure the continuity of services and will further provide support to health care workers in the field and increase their confidence [33, 34]. Some of the limitations of our study is the use of routine immunization administrative coverage data for the period January - August 2019 and 2020, which does not give true coverage as compared to a more recognised WHO/UNICEF estimate or community-based surveys. We also use Penta-3 and MCV1, which are widely used to measure the performance of the immunization system. Our analysis also considers the period of January to August 2019 and 2020, whereas the COVID-19 pandemic started around March for all the countries in the region. Also, we have noted the differences of the cadres of the staff supervised may be different across different facility, district and states. Hence, the need for further studies to consider the cadres of health workers supervised, their years of service, as well as their training level on the job.

## Conclusion

The implementation of integrated supportive supervision (ISS) in the context of COVID-19 pandemic during the period January - August 2020 using the open data kit (ODK) that provides real time value has had varying level of effects by countries in the sub-region, based on the number of visits, the process indicators that are being monitored as well as the outcome indicator (coverage). There was a general decline in most countries on the number of visits conducted, which influenced the indicators that are associated with the visits in some countries. It is therefore important for countries to monitor closely the trend in the number of supportive supervision visits and the frequency at which the visits are conducted, they are also encouraged to explore other opportunities of implementing these visits through piloting the remote supportive supervision based on their peculiarity and feasibility towards ensuring that health workers in the field are provided with the necessary support they require to maintain surveillance and immunization services.

### What is known about this topic

- Supportive supervision using the right tool build health workers confidence and improved immunization performance, and it is also seen as an efficient tool for strengthening health systems;
- The COVID-19 pandemic had caused disruption to basic health services including immunization and vaccine preventable diseases (VPD) surveillance in so many countries of the world;
- Government and WHO, with other partners in the East and Southern African countries are implementing an integrated supportive supervision approach within the Expanded Programme on Immunization (EPI) using mobile data collection to monitor the performance of the system.

### What this study adds

- The COVID-19 pandemic has influenced the conduct of integrated supportive supervision (ISS) visits in most countries of the East and Southern Africa;
- The study identifies that some countries have experienced drop in the number of visits and that has affected the performance of the immunization systems using real time data for action by management team using the ODK platform;
- The study has indicated the need for countries to explore other options of conducting supportive supervision like the remote supportive supervision based on their peculiarity and its feasibility.
